# Seasonal pattern in elderly hospitalized with acute kidney injury: a retrospective nationwide study in Italy

**DOI:** 10.1007/s11255-022-03271-9

**Published:** 2022-07-02

**Authors:** Alfredo De Giorgi, Alda Storari, Pedro Manuel Rodríguez-Muñoz, Rosaria Cappadona, Nicola Lamberti, Fabio Manfredini, Pablo Jesús López-Soto, Roberto Manfredini, Fabio Fabbian

**Affiliations:** 1 Clinica Medica Unit, University Hospital of Ferrara, Ferrara, Italy; 2 Nephrology and Dialysis Unit, University Hospital of Ferrara, Ferrara, Italy; 3 Department of Nursing and Physiotherapy, Universidad de Salamanca, Salamanca, Spain; 4 Department of Nursing, Instituto Maimónides de Investigación Biomédica de Córdoba, Córdoba, Spain; 5 Department of Medical Science, University of Ferrara, Via Luigi Borsari 46, 44121 Ferrara, Italy; 6 Department of Neuroscience and Rehabilitation, University of Ferrara, Ferrara, Italy; 7 Department of Nursing, Universidad de Córdoba, Córdoba, Spain; 8 Hospital Universitario Reina Sofía de Córdoba, Córdoba, Spain

**Keywords:** Acute kidney injury, Elderly, Seasons, Comorbidity, In-hospital mortality, Chronobiology phenomena

## Abstract

**Purpose:**

Acute kidney injury (AKI) frequently complicates hospitalization and is associated with in-hospital mortality (IHM). It has been reported a seasonal trend in different clinical conditions. The aim of this study was to evaluate the possible relationship between seasons of the year and IHM in elderly hospitalized patients with AKI.

**Methods:**

We selected all admissions complicated by AKI between 2000 and 2015 recorded in the Italian National Hospital Database. ICD-9-CM code 584.xx identified subjects with age ≥ 65 years and age, sex, comorbidity burden, need of dialysis treatment and IHM were compared in hospitalizations recorded during the four seasons. Moreover, we plotted the AKI observed/expected ratio and percentage of mortality during the study period.

**Results:**

We evaluated 759,720 AKI hospitalizations (mean age 80.5 ± 7.8 years, 52.2% males). Patients hospitalized with AKI during winter months had higher age, prevalence of dialysis-dependent AKI, and number of deceased patients. In whole population IHM was higher in winter and lower in summer, while the AKI observed/expected ratio demonstrated two peaks, one in summer and one in winter. Logistic regression analysis demonstrated that parameters such as age, autumn, winter, comorbidity burden were positively associated with IHM.

**Conclusion:**

We conclude that a seasonality exists in AKI, however, relationship between seasons and AKI could vary depending on the aspects considered. Both autumn and winter months are independent risk factors for IHM in patients with AKI regardless of age, sex and comorbidity burden. On the contrary, summer time reduces the risk of death during hospitalizations with AKI.

## Introduction

To establish effective public health interventions, proper evaluation of seasonally fluctuating factors is important. Environmental factors such as temperature, humidity, indoor activity, infections should be taken into account in elderly to plan prevention and educational strategies. Therefore, the identification of diseases seasonality could help health care professionals in planning preventive measures, developing effective policies and allowing for use resources more efficiently and effectively [1].

In Hippocratic writings, it is stated that diseases could be correlated with seasons or weather conditions, and mortality shows seasonal fluctuations with winter peak, suggesting that increased mortality in cooler months has been known since 400 B.C. [2].

Several epidemiological studies have reported a seasonal trend in different clinical conditions in the surgical [3], traumatological [4] and medical [5] fields, in particular cardiovascular [6–12], respiratory [13] and infectious illness [14] have got a winter peak in their frequency. Also, acute kidney injury (AKI) has been described to have an incidence peak during winter [15, 16].

AKI has also been reported as a frequent condition among the elderly population and recognizes several risk factors such as advanced age, sepsis, surgery and comorbidities, including systemic arterial hypertension, diabetes mellitus, heart disease, neoplasms and chronic kidney disease [17]. Furthermore, AKI is a frequent finding in hospitalized individuals complicating the clinical course of patients and AKI is associated with higher in-hospital mortality (IHM) and use of resources [18].

Despite all the recent technical and therapeutic advances, the overall mortality of AKI patients remains high, reaching 80% in ICU patients. We previously evaluated the association between AKI and IHM in a large nationwide cohort of elderly subjects in Italy and found that the increasing burden of comorbidity, dialysis-dependent AKI, and sepsis were the major risk factors for IHM [19]. Moreover, we analyzed the relationship between time of the week and IHM in elderly Italian patients admitted for AKI. Our results showed that subjects admitted during weekend with AKI are exposed to a higher risk of IHM, especially if they need dialysis treatment and have high comorbidity burden [20].

The aim of this study was to evaluate the possible relationship between seasons of the year and IHM in elderly patients discharged with AKI diagnosis recorded in the Italian National Hospital Database.

## Materials and methods

### Patient selection and eligibility

The design of this study is retrospective, conducted in agreement with the Declaration of Helsinki of 1975, revised in 2013. Subjects could not be identified, personal data were deleted before analysis aiming at maintaining data anonymity and confidentiality. Ethics committee approval was not required because the study was conducted in agreement with the existent Italian disposition-by-law (G.U. n.76, 31/03/2008).

The Italian Ministry of Health (General Directorate for Health Planning) allowed to access the National Hospital Database (NHD), where all hospitalizations, both in public and private Italian hospitals, codified as discharge hospital records (DHR), are recorded. We selected all admissions complicated by AKI between December 22, 2000, and December 21, 2015. The DHR files contain information such as sex, age, date and department of admission and discharge, vital status at discharge (in-hospital death vs. discharged alive), main diagnosis, up to five comorbidities, and up to six procedures/interventions. Diagnoses and procedures are classified on the basis of the International Classification of Diseases, 9th Revision, Clinical modification (ICD-9-CM). Following the national disposition-by-law in terms of privacy, personal data and all other potential identifiers were removed from the database by the Ministry of Health. The only identifier was a consecutive number for each patient.

In administrative database codes, acute renal failure was usually the reference definition, although in clinical settings AKI replaced the term acute renal failure. Patients aged ≥ 65 years identified by the ICD-9-CM code 584.xx as a first or second discharge diagnosis were selected. The time period was divided into four 3-month intervals depending on the season, i.e., spring (21 March–20 June), summer (21 June–22 September), autumn (23 September–21 December), and winter (22 December–20 January). According to the Koppen-Geiger climate classification scheme, Italian climate could be considered as Cfb (warm temperate, fully humid, warm summer) in the north and Csa (warm temperate, summer dry, hot summer) in the south [21].

### Data analysis

We evaluated IHM related to the season of hospitalization. We also evaluated the comorbidity burden, using a modified Elixhauser Index (mEI) [22, 23]. It was calculated based on the guidelines set by Quan et al. [24]. To calculate the score, the following conditions, based on administrative data, were considered: age, sex, presence of chronic kidney disease (CKD), neurological disorders, lymphoma, solid tumor with metastasis, ischemic heart disease, congestive heart disease, coagulopathy, fluid and electrolyte disorders, liver disease, weight loss, and metastatic cancer. The original score was corrected, removing the diagnosis of previous AKI; therefore, the points assigned to renal diseases were considered only if CKD was recorded. The score was calculated automatically. Finally, we also considered dialysis treatment (code ICD-9-CM 39.95).

### Statistical analysis

A descriptive analysis of the whole population was performed and the data were expressed as absolute numbers, percentages, and means ± SD. Univariate analysis was carried out by using the Chi-Squared, ANOVA or Kruskal–Wallis test as appropriate, comparing age, sex, length of stay, comorbidity burden, dialysis-dependent AKI and mortality during the four seasons of the year. Moreover, to evaluate the relationship between seasonality and IHM, the latter was considered as the dependent variable in a logistic regression analysis, while demography, seasons (spring as the reference), comorbidity score, and dialysis-dependent AKI were considered as independent variables. Odds ratios (ORs) with their 95% confidence intervals (95% CI) were reported.

Moreover, we plotted the AKI observed/expected ratio and percentage of mortality during the study period. The observed numbers of AKI events per month were calculated as the monthly numbers of events over the whole study period. The expected numbers of cases per month were obtained by dividing the average total numbers of patients per year by 365.25, and by multiplying the results by the numbers of days in each month, considering 28.25 days for February. The AKI observed/expected ratio of events was, thus, calculated. All *p* values were two tailed, and *p* value < 0.05 was considered significant. Statistical product and service solution (SPSS) 23.0 for Windows (IBM Corp., Armonk, NY, USA) was used for the statistical analyses.

## Results

We considered a 15-year period (2001–2015), and evaluated 759,720 AKI hospitalizations of whom 52.2% were males. Mean age of the population was 80.5 ± 7.8 years, mean length of stay was 13.7 ± 15.5 days, and dialysis-dependent AKI cases were 68,561 (9%). Mean comorbidity burden calculated by the score was 14.57 ± 6.21. AKI hospitalizations during spring were 187,128 (24.6%), during summer 197,373 (26%), during autumn 181,198 (23.9%), and winter hospitalizations 194,021 (25.5%). Deceased subjects during admissions were 210,305 (27.7%) (Table 1).

**Table 1:** Demographic and clinical characteristics of the evaluated population

Number of records	759,720
Male (*n* (%))	396,653 (52.2%)
Female (*n* (%))	363,067 (47.8%)
Spring hospitalizations (*n* (%))	187,128 (24.6%)
Summer hospitalizations (*n* (%))	197,373 (26%)
Autumn hospitalizations (*n* (%))	181,198 (23.9%)
Winter hospitalizations (*n* (%))	194,021 (25.5%)
Deceased (*n* (%))	210,305 (27.7%)
Dialysis-dependent AKI (*n* (%))	68,561 (9%)
Age (years)	80.5 ± 7.8
Length of stay (days)	13.7 ± 15.5
modified Elixhauser Index	14.57 ± 6.21

*AKI* acute kidney injury

Table 2 shows the seasonal differences of the different characteristics analyzed both on the whole population and in the subgroups of patients with non-dialysis-dependent AKI and dialysis-dependent AKI. Males had higher prevalence during all seasons. Patients hospitalized with AKI during winter months had higher prevalence of dialysis-dependent AKI, higher number of deceased patients and were older. The difference in comorbidity burden, although statistically significant, could not be considered clinically impacting. The same characteristics were showed in the subgroup of patients with non-dialysis-dependent AKI, whilst in dialysis-dependent AKI population, IHM and length of stay were higher during fall. In the same way, the difference in comorbidity burden, although statistically significant, could not be considered clinically evaluable.

**Table 2:** Comparison of the demographic and the clinical parameters in the patients hospitalized in the four different seasons of the year

Whole population	Spring hospitalizations (*n* = 187,128)	Summer hospitalizations (*n* = 197,373)	Autumn hospitalizations (*n* = 181,198)	Winter hospitalizations (*n* = 194,021)	*p*
Male (*n* (%))	97,498 (52.1%)	102,052 (51.7%)	95,742 (52.8%)	101,361 (52.2%)	< 0.001
Female (*n* (%))	89,630 (47.9%)	95,321 (48.3%)	85,456 (47.2%)	92,660 (47.8%)	
Dialysis-dependent AKI (*n* (%))	17,218 (9.2%)	15,667 (7.9%)	16,920 (9.3%)	18,756 (9.7%)	< 0.001
Age (years)	80.4 ± 7.8	80.5 ± 7.8	80.5 ± 7.8	80.7 ± 7.8	< 0.001
modified Elixhauser Index	14.47 ± 6.19	14.62 ± 6.27	14.58 ± 6.21	14.62 ± 6.18	< 0.001
Length of stay (days)	13.87 ± 15.17	13.07 ± 13.84	13.75 ± 14.15	14.15 ± 18.21	< 0.001
Deceased (*n* (%))	50,457 (27%)	49,393 (25%)	51,807 (28.6%)	58,648 (30.2%)	< 0.001

Dialysis-dependent AKI	Spring hospitalizations (*n* = 17,218)	Summer hospitalizations (*n* = 15,667)	Autumn hospitalizations (*n* = 16,920)	Winter hospitalizations (*n* = 18,756)	*p*
Male (*n* (%))	9770 (56.7%)	9038 (57.7%)	9592 (56.7%)	10,738 (57.3%)	0.218
Female (*n* (%))	7448 (43.3%)	6629 (42.3%)	7328 (43.3%)	8018 (42.7%)	
Age (years)	77 ± 6.8	76.8 ± 6.8	77.1 ± 6.8	77.2 ± 6.8	< 0.001
modified Elixhauser Index	12.59 ± 5.54	12.57 ± 5.54	12.76 ± 5.58	12.76 ± 5.56	0.007
Length of stay (days)	21.8 ± 19.9	21.2 ± 19.4	21.7 ± 19.8	21.5 ± 19.9	0.01
Deceased (*n* (%))	7625 (44.3%)	6925 (44.2%)	7854 (46.4%)	8604 (45.9%)	< 0.001

Non-dialysis-dependent AKI	Spring hospitalizations (*n* = 169,910)	Summer hospitalizations (*n* = 181,706)	Autumn hospitalizations (*n* = 164,278)	Winter hospitalizations (*n* = 175,265)	*p*
Male (*n* (%))	87,728 (51.6%)	93,014 (51.2%)	86,150 (52.4%)	90,623 (51.7%)	< 0.001
Female (*n* (%))	82,181 (48.4%)	88,692 (48.8%)	78,128 (47.6%)	84,642 (48.3%)	
Age (years)	80.8 ± 7.8	80.8 ± 7.8	80.9 ± 7.8	81.1 ± 7.8	< 0.001
modified Elixhauser Index	14.66 ± 6.22	14.80 ± 6.29	14.78 ± 6.24	14.82 ± 6.20	< 0.001
Length of stay (days)	13.1 ± 14.4	12.3 ± 13	12.9 ± 13.2	13.4 ± 17.8	< 0.001
Deceased (*n* (%))	42,832 (25.2%)	42,468 (23.4%)	43,953 (26.8%)	50,044 (28.6%)	< 0.001

Comparison is reported in the whole population, in the dialysis-dependent AKI group and in the non-dialysis-dependent AKI group

*AKI* acute kidney injury

Logistic regression analysis (Table 3) demonstrates that age, autumn, winter, comorbidity burden are positively associated with IHM in all groups considered. Summer appears to reduce the risk of death during hospitalization, on the contrary, males have higher risk of death during hospitalization in the dialysis-dependent AKI group, whilst females have higher risk of IHM in the whole population and in the non-dialysis-dependent AKI group.

**Table 3:** Logistic regression analysis of factors independently associated with in-hospital mortality in the whole population, in the dialysis-dependent AKI group and in the non-dialysis-dependent AKI group

Whole population	OR	95% CI	*p*
Spring hospitalizations	Ref		
Summer hospitalizations	0.907	0.893–0.920	< 0.001
Autumn hospitalizations	1.080	1.064–1.096	< 0.001
Winter hospitalizations	1.162	1.145–1.178	< 0.001
Dialysis-dependent AKI	2.879	2.832–2.927	< 0.001
modified Elixhauser Index	1.043	1.042–1.044	< 0.001
Age	1.022	1.021–1.023	< 0.001
Female sex	1.022	1.011–1.033	< 0.001

Dialysis-dependent AKI	OR	95% CI	*p*
Spring hospitalizations	Ref		
Summer hospitalizations	0.996	0.954–1.041	0.871
Autumn hospitalizations	1.089	1.044–1.137	< 0.001
Winter hospitalizations	1.064	1.020–1.109	0.004
modified Elixhauser Index	1.009	1.006–1.012	< 0.001
Age	1.004	1.001–1.007	0.003
Male sex	1.137	1.102–1.173	< 0.001

Non-dialysis-dependent AKI	OR	95% CI	*p*
Spring hospitalizations	Ref		
Summer hospitalizations	0.895	0.881–0.909	< 0.001
Autumn hospitalizations	1.079	1.062–1.096	< 0.001
Winter hospitalizations	1.176	1.158–1.194	< 0.001
modified Elixhauser Index	1.047	1.046–1.048	< 0.001
Age	1.024	1.023–1.025	< 0.001
Female sex	1.037	1.025–1.049	< 0.001

*AKI* acute kidney injury

**Fig. 1:**
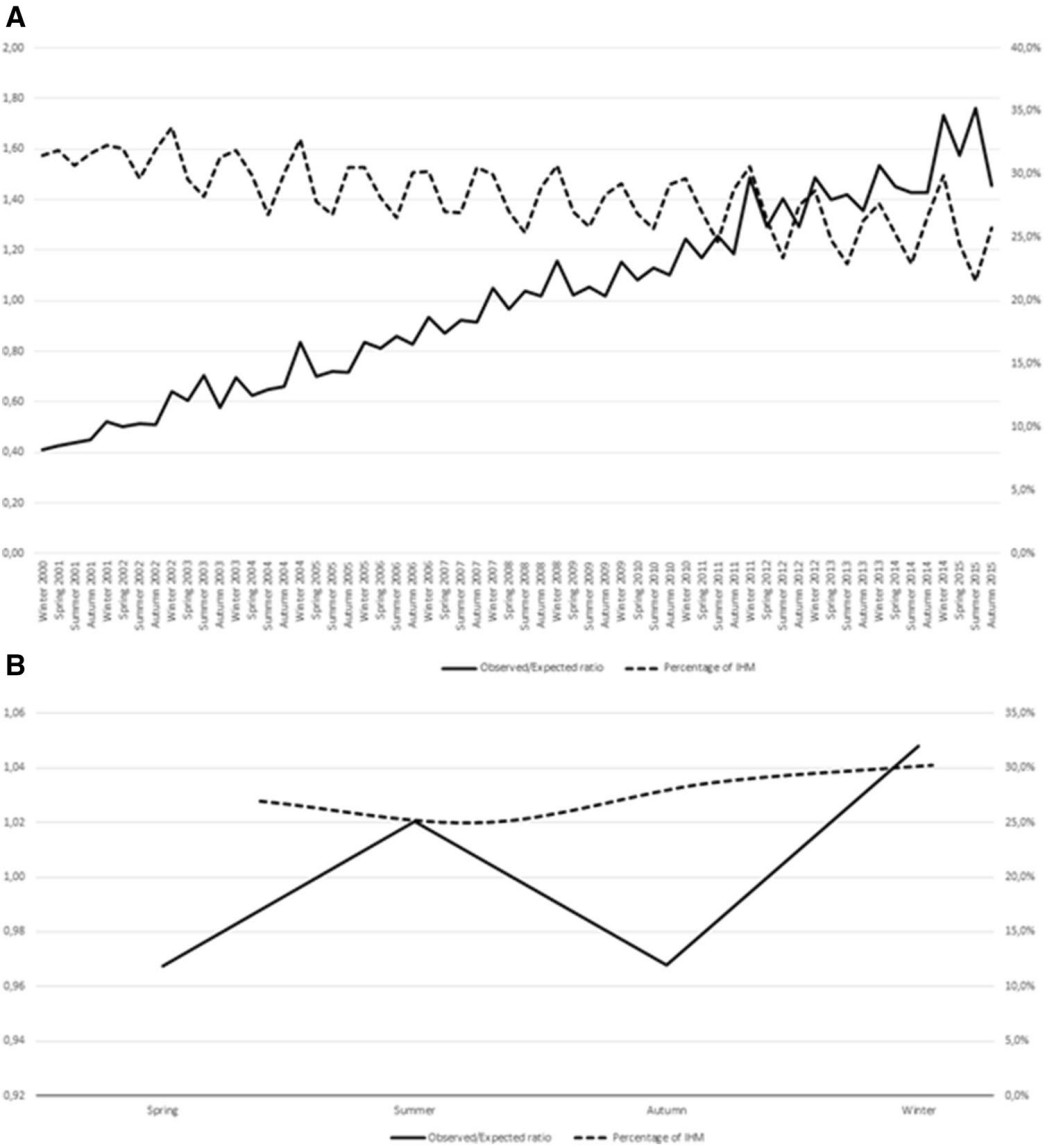
The AKI observed/expected ratio and percentage of mortality during hospitalization during the 15-year period in the whole population, considering all seasons throughout the period of the study (**A**) and during the four seasons including all years (**B**)

**Fig. 2:**
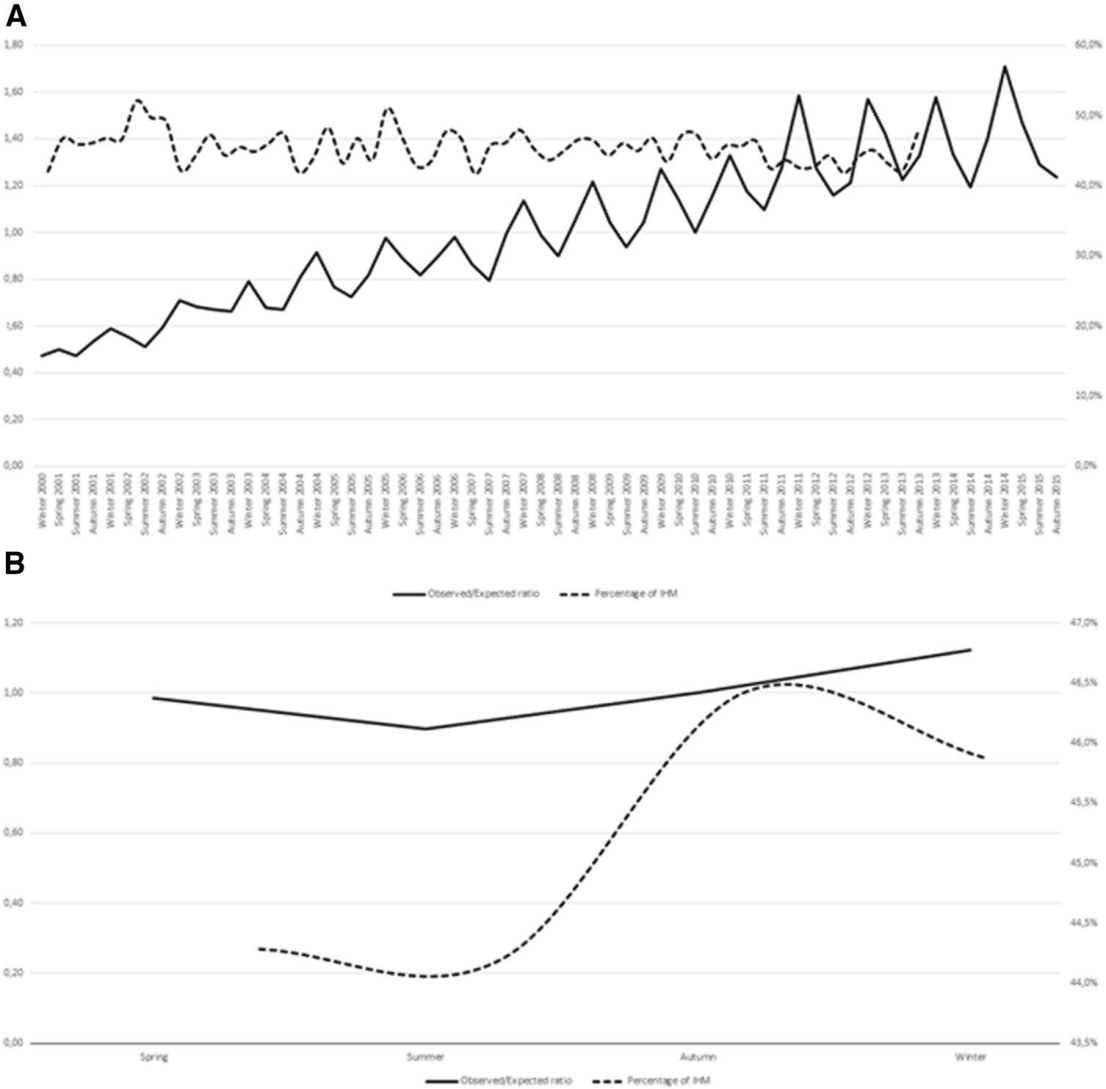
The AKI observed/expected ratio and percentage of mortality during hospitalization during the 15-year period in the dialysis-dependent AKI group, considering all seasons throughout the period of the study (**A**) and during the four seasons including all years (**B**)

Figure 1 shows the AKI observed/expected ratio and percentage of mortality during hospitalization during the 15-year period in the whole population, considering all seasons throughout the period of the study (A) and during the four seasons including all years (b). In whole population IHM is higher in winter and lower in summer, while the AKI observed/expected ratio considering the four seasons including all years demonstrates two peaks, one in summer and one in winter. Similar patterns are calculated in non-dialysis-dependent AKI (data not shown); whilst in dialysis-dependent AKI group, a seasonal periodicity pattern is evident only for the AKI observed/expected ratio considering all seasons throughout the period of the study. Evaluation of percentage of IHM considering the four seasons including all years shows a peak in autumn (Fig. 2).

## Discussion

In this study, we found that the highest number of hospitalizations with AKI is detected during summer; however, admitted subjects suffering AKI have higher winter mortality associated with higher prevalence of dialysis-dependent AKI and higher age; on the contrary, patients suffering dialysis-dependent AKI have higher mortality during Autumn. Therefore, despite a higher frequency of AKI, summer seems to reduce the risk of IHM in both non-dialysis and dialysis-dependent AKI populations, whilst autumn and winter seem to increase the risk of IHM in the two groups, as well as age and comorbidity burden. IHM has a winter peak in the whole population, whist the AKI observed/expected ratio has two peaks, in summer and winter in the whole population, but in the dialysis-dependent AKI group we see only a winter peak. In this population IHM has a peak in autumn.

Particularly interesting is the fact that male sex increases the risk of IHM in dialysis-dependent AKI population, while female sex increases the risk of IHM in non-dialysis-dependent AKI. However, it is somewhat difficult to explain this finding.

To the best of our knowledge, this is the first study considering the relationship between seasons of the year and both AKI observed/expected ration and IHM in AKI associated admissions in Italy. The relationship between seasonality and renal diseases is matter of debate and results from different studies could be interpreted as contradictory, probably depending on different populations selection, outcome, study design, and degree of renal impairment. In fact, results of different studies are different depending also on evaluation of AKI incidence or mortality.

Seasonal mortality pattern has been described in non-human primates influenced both by diet and degree of environmental seasonality [25]. Similarly to what happens in non-human primates, the relationship between seasonality and mortality in human societies should take into considerations several confounding factors, including social factors.

Indeed, in New Zealand, a temperate country, winter mortality was higher among low-income people, those living in rented accommodation and those living in cities [26]. In the same country excess of winter mortality was found to be 18% higher than expected from non-winter months [27]. Mortality from diseases of the respiratory and circulatory system was most dependent on seasonal effect. Also pathological conditions involving the blood, the endocrine system and metabolism, and diseases of the digestive system had rates of death in winter and non-winter month ratio higher than one. Similar results were also reported in less temperate areas such as Pakistan, where patients aged more than 54 years had high risk of death during the winter season as compared to the summer season, especially if they suffered from cardiovascular, respiratory and kidney diseases. On the other hand, authors underlined that the majority of people living in the analyzed region had socio-economics problem such as a very low standard of living and low quality houses residence [28]. It has also underlined that females could be more vulnerable than males to winter phenomenon [27]. In our study women had higher risk for IHM if AKI was not treated with dialysis. A review published in 2015 established that the elderly, children, and males could be considered the vulnerable populations during heat waves, demanding increased medical care especially in the presence of existing chronic diseases. Moreover, social factors, such as lower socio-economic status, could contribute to heat susceptibility. Authors suggested that relevant policies and guidelines should be developed to protect vulnerable populations, adopting morbidity indicators during heat wave early warning systems to ameliorate public health actions [29].

Climate impacts health, it has been established that global warming may influence human health [30], and increasing temperature impact health status [31]. Data from Adelaide, South Australia, established that augmentation in daily temperature of 1 °C was associated with an increased incidence for different renal disease such as urolithiasis, AKI, chronic kidney disease (CKD), urinary tract infections (UTIs), and lower urinary tract infections (LUTIs) [32]. A Korean research group reported increasing AKI admissions by 23.3% per 1 °C rise in mean temperature above the 28.8 °C flexion point in the warm season [33]. The cut-off for an increased risk of AKI has been reported to be 22.3 °C with a high correlation between ambient temperature and emergency department visit for AKI [34]. In our study, the AKI observed/expected ratio in the whole population and in the non-dialysis-dependent AKI group has a peak in summer as well as in winter time. However, this seasonal behavior appears to belong to AKI with less degree of renal impairment, in fact IHM and the AKI observed/expected ratio of hospitalization in dialysis-dependent AKI group are higher in winter. The frequency of AKI requiring only medical treatment is high especially during summer months. It was found that in patients with a median age of 80 years with high prevalence of hypertension, diabetes, cardiovascular disease and chronic liver disease, environmental heat increased the risk of AKI by 11%. Incidence of AKI during warm months was 182 cases per 100,000 person-years [35]. Similar results were reported by Bobb et al. [36] that identified causes of hospital admissions during extreme heat events evaluating 23.7 million fee-for-service beneficiaries, aged ≥ 65 years, during the period 1999–2010 with at least five summers of near-complete daily temperature data in the United States. They found that risks of hospitalization for fluid and electrolyte disorders, renal failure, UTIs, septicaemia, and heat stroke were statistically significantly higher on heat wave days relative to matched non-heat wave days [36]. Also, it has been described that heavy occupational workload in high ambient temperature is associated with acute reduction in kidney function [37]. Moreover, even in hypertensive patients, with and without chronic kidney disease, it has been demonstrated similar seasonal variations in estimated glomerular filtration rate (eGFR) with lower values during summer (June–August) compared with spring (March–May). The decrease in eGFR from spring to summer was similar for both groups, however, the percent change in eGFR was higher in hypertensive patients with renal impairment [38].

Cardiovascular disease (CVD) and renal disease are closely related [39], and a clear seasonal pattern has been reported for CVD, with the highest incidence occurring during the colder winter months [40]. CVD follows a seasonal pattern with a winter peak and clusters of all subtypes of CVD after ‘cold snaps’, and with corollary peaks linked to heat waves [41].

None of the previous studies have considered mortality, while in our study the main outcome is IHM and we take into consideration the observed/expected ratio of AKI needing hospitalization.

A retrospective study by our group, based on the database of hospital admissions of the region Emilia-Romagna of Italy (years 1998–2006), analyzed 64,191 cases of acute myocardial infarction (AMI), of whom 62.9% males, and 12.3% fatal [42]. Acute myocardial infarction was most frequent in winter and least in summer, with the highest number of cases in January and the lowest in July. Chronobiologic analysis showed winter peaks for total cases (January), females (December), and fatal cases (January) [42]. More recently, a study from the United States analyzed nearly 11 × 10^6^ adult admissions for acute myocardial infarction using the National Inpatient Sample (2000–2017). Admissions, happened in 24.3, 22.9, 22.2, and 24.2% in spring, summer, autumn, and winter, respectively. Despite an approximately similar disease incidence, compared to spring, winter admissions had higher IHM risk, whilst summer and autumn had slightly lower IHM risk [43].

All-cause mortality and death due to CVD was higher during the cold months than during the warm ones also in patients receiving dialysis treatment [44, 45]. Dialysis treatment appears to worsen prognosis of patients admitted with AKI, and we find higher IHM in dialysis-dependent AKI group during autumn and winter, suggesting a relationship between dialysis treatment and cold months. In 2012, Usvyat et al. [44] evaluated whether mortality related with seasonal changes was present in high-risk haemodialysis patients. They evaluated more than 15,000 subjects and found that mortality, both all-cause and cardiovascular, was significantly higher during winter compared with other seasons. Seasonal variations were similar across climatologically different regions. Differences in mortality disappeared when adjusted for seasonally variable clinical parameters. Therefore, they concluded that significant seasonal variations in overall and cardiovascular mortality were consistent over different climatic regions, and that mortality differences were related to seasonality of physiologic and laboratory parameters. Higher winter mortality in dialysis patients was confirmed by the International monitoring dialysis outcomes (MONDO) consortium, but it was true only outside the tropical zones [46].

In addition, the analysis of the United States Renal Data System database 2000–2013, showed that transitioning to end-stage renal disease (ESRD) and adverse events, including all-cause, cardiovascular and infectious mortalities, were more frequent in winter and less frequent in summer [47]. In 2020, Goto et al. [48], using the Japanese database for dialysis patients, compared the fractions of all-cause and cause-specific mortality among seasons after adjustment for different variables. They concluded that all-cause mortality, and mortality from coronary heart disease, heart failure, cerebral hemorrhage, and infectious pneumonia were significantly higher in winter than in summer. Although acute aortic dissection exhibits a winter peak too [49], a further analysis on cases enrolled at various sites around the globe, belonging to the International Registry of Acute Aortic Dissection (IRAD), revealed that the winter peak was evident in both cold and temperate climate settings, suggesting that the relative change in temperature, rather than absolute temperature, and/or endogenous annual rhythms could be critical mechanistic factors [50].

Similar seasonal patterns were shown in transplant medicine, although new kidney transplantation was highest in summer, whereas outcome of transplant patients was reported to be worse during winter (January) [47] as well as graft failures due to chronic rejection [51]. Gilbertson et al. [52] evaluated data from the Center for Diseases Control and Prevention (CDC) Outpatient Influenza-like Illness Surveillance network and centers for medicare and medicaid services, and ascribed excess of mortality of patients with end-stage renal disease to serious respiratory tract infections such as influenza-like illness. Indeed, a recent study from Hong Kong revealed a relationship between admissions due to AKI and hospitalization caused by influenza, and the risk was increased significantly by low temperature [53].

Studies investigating AKI found results similar to ours. In 2017, Phillips and colleagues identified the seasonal pattern of incidence and outcome of AKI using the Welsh National Health Service. The highest frequency of both, community-acquired and hospital-acquired AKI, was detected between January and March, whilst 90-day mortality after AKI episode had two peaks, the first one was included between January and March, and the second between October and December [54]. Iwagami et al. [15] evaluated more than 80,000 patients with AKI and found that prevalence of AKI was higher in January than in June, moreover, further evaluations suggested that the seasonality of AKI was related to community-acquired AKI associated with the admission diagnosis of cardiovascular and pulmonary diseases among older patients. Also 30-day mortality was higher in winter months. Similar results were highlighted in an Italian hospital setting, underlining how winter was associated with increased risk of AKI and that low air temperature and high relative humidity increased the risk of AKI [16].

Therefore, populations with low degree of renal impairment appear to have higher risk for hospitalization during warm months, on the contrary, AKI with serious renal damage associated with comorbid burden and the need of dialysis treatment could suffer winter peak regarding hospitalization and IHM.

## Limitations

Retrospective studies, based on administrative data, have major limitations without the possibility to assess the degree of renal impairment and the cause of AKI. Administrative databases do not allow to evaluate some important items, such as cause of admission and death, intensive care level or hospitals’ facilities, device use, type of treatment, and impact of clinical or biochemical parameters. However, we could differentiate the population investigated in non-dialysis-dependent AKI and dialysis-dependent AKI, a classification suggesting the degree of renal impairment. Administrative databases are used for different reasons such as reimbursement, and lack of specific clinical information and could be cause of possible misclassification [55]. Moreover, AKI was not identified on the basis of international Kidney Disease Improving Global Outcomes (KDIGO) guidelines [56], and the cause of AKI, as well as treatment setting were missing, with the exception of dialysis treatment. In the same way we could not consider socio-economic parameters. The performance of ICD-9-CM for diagnosis of AKI has been reported to have poor sensitivity, and high specificity, while positive and negative predictive values could differ [57–59]. However, in subjects aged ≥ 65-year sensitivity significantly increased and administrative data detected more severe AKI and higher IHM mortality [60]. For these reasons, we decided to focus on patients aged > 65 years. Data on weather conditions and environmental factors during the study period were missed, we did not analyze climatic parameters such as temperature and humidity; on the other hand, we reported how Koppen-Geiger classified Italian climate [21]. We should also underline some strengths of our study such as the sample size derived from a national database, the significant period of time considered, and the use of IHM as hard outcome.

## Conclusions

Relationship between AKI, comorbidity and IHM is a very actual debate [61–64]. Our results suggest that also seasonality should be taken into account especially in Italy, a country belonging to warm temperate climates [21]. However, relationship between seasons and AKI could vary depending on the aspect considered, i.e., the AKI observed/expected ratio, IHM or degree of renal impairment. With this study we have tried to stratify an important population to better understand seasonality at a national level. We conclude that both autumn and winter months are independent risk factors for IHM in patients with AKI regardless of age, sex and comorbidity burden, while the risk of death in AKI subjects that require in-hospital treatment is lower during the summertime. Understanding disease seasonality could ameliorate clinical practice, improving hospital resource utilization and community-based preventive care.

## Data Availability

The datasets generated and/or analyzed during the current study are not publicly available due to privacy policy but are available from the corresponding author on reasonable request.
